# Effects of home-based prescribed pulmonary exercise by patients with chronic obstructive pulmonary disease: study protocol for a randomized controlled trial

**DOI:** 10.1186/s13063-018-3149-7

**Published:** 2019-01-11

**Authors:** Xiaodan Liu, Peijun Li, Lu Xiao, Yufan Lu, Ning Li, Zhengrong Wang, Hongxia Duan, Jian Li, Weibing Wu

**Affiliations:** 1 School of Rehabilitation Science, Shanghai University of Traditional Chinese Medicine, Shanghai, China; 2 Institute of Rehabilitation Medicine, Shanghai Academy of Traditional Chinese Medicine, Shanghai, China; 3 Department of Sports Medicine, Shanghai University of Sport, Heng Ren Road No 188, Yang Pu District, Shanghai, 200438 China

**Keywords:** COPD, skeletal muscle function, prescribed pulmonary exercise, resistance exercise, home-based rehabilitation

## Abstract

**Background:**

Chronic obstructive pulmonary disease (COPD) not only affects pulmonary function but also leads to skeletal muscle dysfunction. The various characteristics of different forms of traditional Chinese exercise lead to inconsistent clinical effects in COPD patients. Hence, the present study carefully combined and rearranged *liuzijue*, *wuqinxi*, *baduanjin*, and *yijinjing* into a pulmonary exercise program targeting COPD patients.

**Methods/design:**

This study is a single-blind, randomized controlled trial. A random number table will be generated by an independent person. Each number will be placed in a sealed opaque envelop to blind assignment. All outcome assessors will be blinded to group assignment.

COPD patients between 40 and 80 years of age, with stable medical treatment and no regular participation in regular exercise in the last 6 months will be included. All participants will be recruited from the Respiratory Medicine Department of Yue-Yang Integrative Medicine Hospital Affiliated to Shanghai University of Traditional Chinese Medicine.

All participants will continue to follow their medical treatment. They will be randomly assigned to one of four groups in a 1:1:1:1 ratio: (1) usual care (control group, CG), (2) pulmonary exercise group (PG), (3) resistance exercise group (RG), or (4) combined pulmonary exercise and resistance exercise group (PRG). CG participants will receive medical treatment only. PG participants will perform 60 min of exercise twice a day 7 days a week for 3 months, with 1 day’s exercise per week at hospital under guidance and supervision. RG participants will perform 60 min of resistance exercise once a day, three times a week for 3 months, with 1 day’s exercise per week at hospital under guidance and supervision. PRG participants will perform 60 min of prescribed pulmonary exercise combined with resistance exercise for 3 months.

The outcomes include the isokinetic strength of peripheral skeletal muscle, surface electromyography, 6-min walking distance, 30-s arm curl test, pulmonary function, respiratory muscle strength, dyspnea, body composition, physical activity, quality of life, and Chronic Disease Self-Efficacy Scale.

**Discussion:**

The results of this study will compensate for the current inadequate understanding of prescribed pulmonary exercise and may provide a new, simple, convenient, and effective home-based exercise intervention for COPD patients.

**Trial registration:**

Chinese Clinical Trial Registry, ChiCTR-1800017405. Registered on 28 July 2018.

**Electronic supplementary material:**

The online version of this article (10.1186/s13063-018-3149-7) contains supplementary material, which is available to authorized users.

## Background

Chronic obstructive pulmonary disease (COPD) is a chronic respiratory disease characterized by persistent and progressive airflow limitation, which increases morbidity and mortality. According to statistics reported in *The Lancet*, the morbidity in Chinese people over the age of 40 years has increased from 8.2% in 2008 to 13.7% in 2015 [1], and worldwide mortality from COPD increased by 11.6% in 2015 compared to 1990 [2]. As a systemic disease, COPD not only affects the lungs, causing dyspnea and impaired lung function, but also causes systemic effects including skeletal muscle dysfunction, which further leads to impaired exercise capacity, decreased physical activity, and a poorer quality of life.

Pulmonary rehabilitation (PR) is an important constituent of the non-pharmacological treatment of people with COPD, including but not limited to exercise training, education, and self-behavioral management. It has had significant effects in improving symptoms, exercise capacity, quality of life, and prognosis [3, 4]. Among the diverse methods used in PR, Chinese traditional exercise is a self-healing, functionally integrated aerobic exercise, inducing a possible maximum heart rate of 43–49% predicted [5] and consuming 1.5–2.6 metabolic equivalent units [6]. It is potentially suitable for COPD patients to use at home [7]. The findings from previous studies suggest that Chinese traditional exercise, including tai chi, *liuzijue*, *wuqinxi*, and *yijinjing*, by COPD patients has achieved positive results. Home-based *liuzijue* significantly improved pulmonary function (forced expiratory volume in 1 s [FEV1]), special airway conduction, exercise capacity (measured by the 6-min walking test [6MWT] and 30-s sit-to-stand test), quality of life, and mental health of COPD patients [8, 9]. Moreover, 6 months of home-based (individual) combined with clinic-based (group) *yijinjing* significantly improved pulmonary function (FEV1, FEV1 / forced vital capacity [FVC], and FEV1%pred [FEV1 expressed as a percentage of the predicted value]), exercise capacity (6MWT), quality of life, and emotional regulation in COPD patients [10]. Six months of home-based *baduanjin* significantly improved exercise capacity (6MWT) and quality of life in COPD patients [11]. However, these studies have focused on a single type of traditional Chinese exercise as the intervention, with inconsistent clinical effects on COPD patients according to the exercise type. Moreover, less attention has been paid to the effect of such exercises on skeletal muscle function and physical activity.

**Fig. 1:**
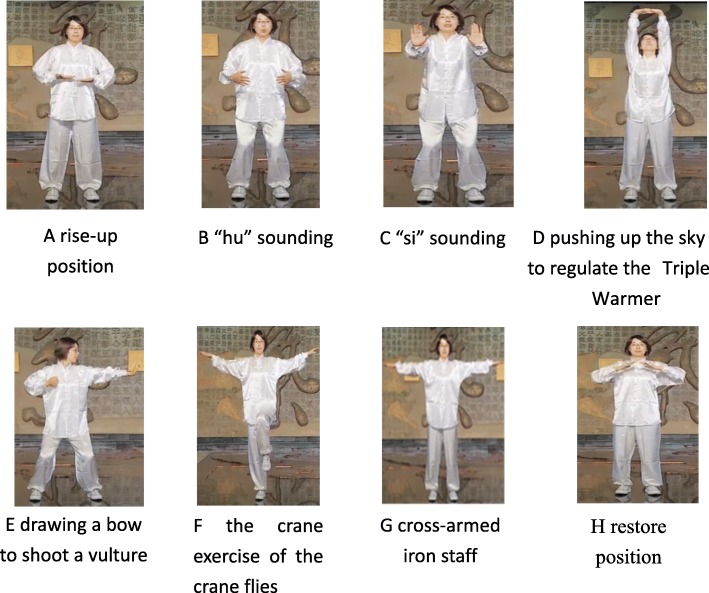
Main characteristics of pulmonary exercise. The picture is from the “Teaching video of COPD patient pulmonary exercise” self-recorded by the research group. The teacher in the video is Xiaodan Liu, a member of the research group. (a) Rise-up position. (b) “hu” sound. (c) “si” sound. (d) Pushing up the sky to regulate the triple warmer. (e) Drawing a bow to shoot a vulture. (f) Crane actions, including the crane spreading its wings and flying. (g) Cross-armed iron staff. (h) Restore position

Following the basic theory of traditional Chinese medicine, the study combined and reorganized elements from *liuzijue*, *wuqinxi*, *baduanjin*, and *yijinjing* to compile a new intervention of prescribed pulmonary exercise for COPD rehabilitation. The main characteristics of prescribed pulmonary exercise include the “hu” and “si” sounds in *liuzijue*; “pushing up the sky to regulate the triple warmer (meridian)” and “drawing a bow to shoot a vulture” in *baduanjin*; “the crane actions, including the crane spreading its wings and flying” in *wuqinxi*; and “cross-armed iron staff” in *yijinjing* (Fig. 1). Liu et al. [12] demonstrated that rearranged traditional Chinese exercises (including “Wei Tuo offers an iron staff,” form 1 and 2 in *yijinjing*; “pushing up the sky to regulate the triple warmer” and “drawing a bow to shoot a vulture” in *baduanjin*; “si,” “hu,” and “chui” sounds in *liuzijue*; and “the crane actions, including the crane spreading its wings and flying” in *wuqinxi*) improved exercise capacity significantly and induced additional benefits in daily living activity and social participation compared with conventional PR (including pursed-lip breathing and aerobic exercises). Consistent with previous studies, Liu’s study did not examine the effects of exercise on skeletal muscle function and physical activity in COPD patients.

However, skeletal muscle dysfunction as a significant extrapulmonary effect of COPD may occur before respiratory symptoms, affecting exercise capacity, physical activity, and quality of life, and is an independent risk factor for predicting mortality in COPD patients [13, 14]. Moreover, exercise limitations in COPD patients may be mainly due to peripheral factors rather than ventilation limitation, and the correlation between lung capacity and exercise capacity is poor [15, 16]. Hence, skeletal muscle function has become an important target for improving the exercise capacity and quality of life of COPD patients and has received increasing attention from clinical and scientific researchers. As an important method for counterbalancing skeletal muscle dysfunction, resistance exercise has obvious advantages in improving skeletal muscle strength and function of COPD patients [17, 18]. Common methods of providing resistance include weight-lifting (dumbbells), bodyweight-lifting, and using elastic bands.

As an easy-to-use, safe, and affordable item, resistance bands are available to most COPD patients and can be applied for home-based exercises. Previous studies have found that the effects of exercising with resistance bands on improving skeletal muscle strength and quality of life in patients with COPD were similar to those of conventional resistance exercise, with the effects on functional exercise capacity (measured by 6MWT) significantly greater than conventional resistance exercise [19]. Exercising with elastic bands has significant positive effects on the endurance of shoulder joints, the strength and endurance of knee joints (measured by the isokinetic strength test), and functional exercise capacity (assessed by 6MWT and 6-min pegboard and ring test) [20]. In addition, home-based long-term exercise with elastic bands can significantly improve skeletal muscle function of the lower extremities (assessed by the isokinetic strength test) and exercise capacity (6MWT and five repetitions of the sit-to-stand test) in COPD patients, and the effects on improving muscle strength (isokinetic peak torque divided by body weight) were more significant than conventional PR [21]. Another study similarly found that home-based long-term exercise with elastic bands can significantly improve the extensor strength of knee joints (assessed by hand-held dynamometry) [22]. Hence, exercising with elastic bands has a wide potential in home-based rehabilitation programs for COPD patients for improving skeletal muscle function and exercise capacity. However, the effects of exercising with resistance bands and the role of prescribed pulmonary exercise on improving skeletal muscle function in COPD patients is unclear, as is whether prescribed pulmonary exercise combined with exercising with elastic bands can achieve additional benefits compared with a single mode of exercise.

Hence, in this study, 3 months of (1) usual care, (2) prescribed pulmonary exercise, (3) exercising with resistance bands, and (4) prescribed pulmonary exercise combined with exercising with elastic bands will be applied as interventions to COPD patients to evaluate the overall effect of prescribed pulmonary exercise on lung function, exercise capacity, skeletal muscle function, quality of life, and psychological function in patients with COPD. The aim of the study is to further clarify the role of prescribed pulmonary exercise in COPD rehabilitation, since it is a simple, fun, easy to learn, operational, and effective intervention for COPD patients. We hypothesize that prescribed pulmonary exercise can significantly improve pulmonary function, exercise capacity, skeletal muscle function, quality of life, and psychological function, and that combined exercise induces additional benefits in COPD patients compared with single exercise methods.

## Methods/design

### Design

This is a single-blind, randomized controlled clinical trial, to investigate the effects of prescribed pulmonary exercise in COPD patients by comparing the effects of (1) usual care, (2) prescribed pulmonary exercise, (3) exercising with elastic bands, and (4) prescribed pulmonary exercise combined with exercising with resistance bands in COPD patients. Participants will be enrolled from the respiratory medicine department of Yue-Yang Integrative Medicine Hospital Affiliated to Shanghai University of Traditional Chinese Medicine. The study protocol has been registered with the Chinese Clinical Trial Registry (ChiCTR-1800017405), and the study has been approved by the Ethics Committee of Yue-Yang Integrative Medicine Hospital Affiliated with Shanghai University of Traditional Chinese Medicine (Shanghai, China). All participants will provide written informed consent to participate in the study.

**Fig. 2:**
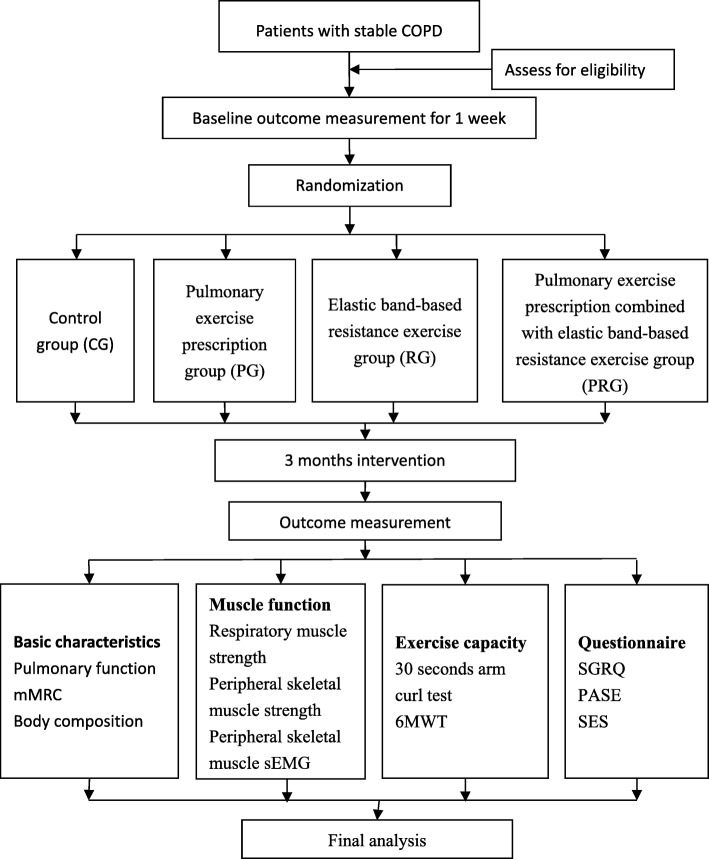
Study flow chart. 6MWT 6-min walking test, CG control group, COPD chronic obstructive pulmonary disease, mMRC Modified Medical Research Council Dyspnea Scale, PASE Physical Activity Scale for the Elderly, PG pulmonary exercise group, PRG pulmonary exercise and resistance exercise group, RG resistance exercise group, sEMG surface electromyography, SES Chronic Disease Self-Efficacy Scale, SGRQ St. George’s Respiratory Questionnaire

**Fig. 3:**
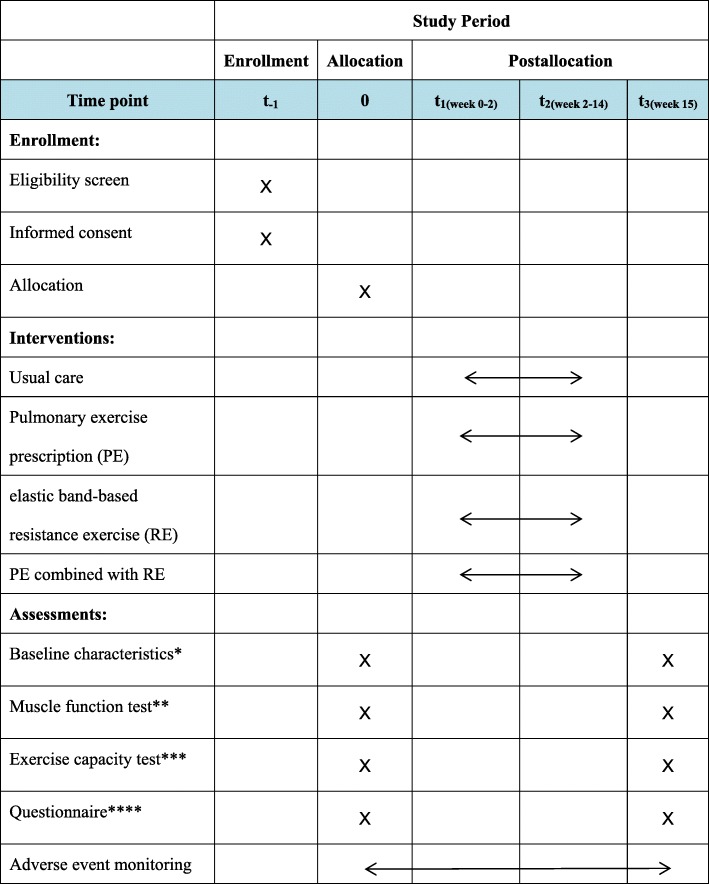
SPIRIT figure showing time points for enrollment, interventions and assessment. PE pulmonary exercise, RE resistance exercise. *Baseline characteristics include pulmonary function assessed by spirometry, dyspnea assessed by the Modified Medical Research Council Dyspnea Scale, body composition assessed by dual-energy X-ray absorptiometry, and height and weight assessed by a body tester. **Muscle function test includes respiratory muscle strength assessed by spirometry, dominant upper and lower limb muscle strength assessed by a CON-TREX isokinetic dynamometer and by a wireless remote-sensing surface electromyography and analysis system. ***Exercise capacity test includes 30-s arm curl test and 6-min walking test. ****Questionnaire includes St. George’s Respiratory Questionnaire, the Physical Activity Scale for the Elderly, and the Chronic Disease Self-Efficacy Scale

Participants will be randomly assigned to one of four groups according to a random number table generated by computer, with randomization conducted by an independent person not participating in the recruitment process. Random numbers will be placed in sealed opaque envelopes to blind the group allocation. As exercise interventions will be used in the study, concealing the allocation to the therapist and participants will not be possible, so only the outcome assessors will be blinded with a new evaluator replacing the evaluator familiar with the allocation, to maintain blinding. The study procedure is shown in Fig. 2 and the time points of the study in Fig. 3. The protocol follows the SPIRIT 2013 checklist, provided in Additional file 1.

### Sample size

The sample size was based on detecting a minimum difference of 54 m in the 6MWT [11] between the control group (CG) and the pulmonary exercise group (PG) and used a baseline standard deviation of 57 m [11]. With a power of 80% and an alpha of 5% (two-sided), the minimum sample size per group is 18 to give a total of 72 participants. Assuming a drop-out rate of 20%, the minimum sample size in total is 88 (22 participants in each group). The participants will be recruited to achieve a balanced number of patients with different levels of severity of COPD.

### Participants

Patients with stable COPD diagnosed at the respiratory medicine department of Yue-Yang Integrative Medicine Hospital Affiliated to Shanghai University of Traditional Chinese Medicine will be invited to participate in the study. Potential participants will be identified from the clinical database and contacted face-to-face or by phone, to assess their basic eligibility for the study. Individuals interested in the study will be scheduled to attend a screening assessment.

The diagnosis of COPD will be confirmed in accordance with the Global Initiative for Chronic Obstructive Lung Disease (GOLD) criteria. Participants will be included if: (1) they have been diagnosed with moderate to very severe COPD (stages II–IV, that is FEV1/FVC < 0.7, FEV1 < 0.8 predicted) [3]; (2) they are between 40 and 80 years of age; (3) they have been clinically stable in the 4 weeks prior to randomization; (4) they have not participated in any organized exercise training (at least twice a week) in the past 6 months; and (5) they are willing to give written informed consent and cooperate accordingly.

The exclusion criteria are: (1) acute exacerbation that requires a change in pharmacological management or hospitalization; (2) coexistence of other chronic respiratory disorders; (3) severe comorbidities including cardiovascular, liver, or kidney disease; (4) skeletal muscle disease or other disease hampering assessment of muscle strength; (5) an open injury affecting the application of surface electromyography (sEMG); or (6) contraindications preventing the assessment of body composition.

### Intervention

All participants will continue with their prescribed medication. They will attend the same weekly educational sessions including on quitting smoking, self-management, and nutrition. In addition, participants in the exercise groups will attend 3 exercise sessions over 2 weeks to become familiar with the intervention programs. During this study time, the participants will be given oral and visual instructions on how to perform the prescribed pulmonary exercises or the exercises with elastic bands and will be given instructions on how to perform the exercises at a Borg CR10 intensity level of between 4 and 6 [23]. In addition, all participants in the exercise groups will receive a teaching video and exercise record book. After each exercise session, participants will be required to record what they have done, including but not limited to time, duration, intensity, and site (home or other).

#### Control group

Participants in the CG will continue with their prescribed medication and will not receive the exercise intervention.

#### Pulmonary exercise group

Participants in the PG will continue with their prescribed medication treatment and receive 3 months of prescribed pulmonary exercise. The PG exercise regimen will comprise sessions twice daily, 7 days per week over 3 months. On Sunday afternoons, the participants will exercise under the supervision and instruction of physiotherapists in a group format. Each of the these sessions will last approximately 60 min and consist of (1) a 10-min warm-up exercise, focusing on the flexibility of the peripheral skeletal muscles; (2) 40 min of prescribed pulmonary exercise consisting of eight characteristics including the “hu” and “si” sounds in *liuzijue*, “pushing up the sky to regulate the triple warmer” and “drawing a bow to shoot a vulture” in *baduanjin*, “the crane actions, including the crane spreading its wings and flying” in *wuqinxi*, and “cross-armed iron staff” in *yijinjing*; and (3) a 10-min cool-down exercise focusing on stretching the peripheral skeletal muscles. Exercise intensity will be self-determined as being between 4 and 6 according to the Borg CR10 scale (meaning patients feel the intensity as somewhat strong to very strong when performing the exercise). Their heart rate will be monitored using a Polar Team^2^ (Polar, Finland).

#### Resistance exercise group

Participants in the RG will continue with their prescribed medication treatment and receive 3 months of exercising with elastic bands. According to previous studies [21, 24], important exercises for the muscle groups of the upper and lower extremities for COPD patients consist of shoulder shrugs (trapezius), straight arm shoulder abductions (deltoid), arm curls (biceps), chest presses (pectoralis major and deltoid anterior), hip abductions (mesoglutaeus), stiff-legged dead lifts (hamstrings), body squats (gluteus maximus and quadriceps), and standing calf raises (gastrocnemius and soleus). These will be performed with elastic bands (Thera-Bands®, Hygenic Corporation, USA) (Table 1). The bands provide a different resistance according to their color: tan (extra thin) < yellow < red < green < blue < black < silver < gold (maximum resistance) [24]. This will allow participants to choose an appropriate exercise intensity when performing the resistance exercise. The RG regimen will comprise three exercise sessions per week over 3 months. On Sunday afternoons, the participants will exercise under the supervision and instruction of physiotherapists in a group format. Each session will span approximately 60 min and consist of (1) a 10-min warm-up exercise focusing on the flexibility of the eight muscle groups; (2) 40 min of exercises with the elastic bands involving the eight muscle groups (8 to 12 repetitions for each muscle group repeated 3 times with 1-min rest intervals); (3) a 10-min cool-down exercise focusing on stretching the eight muscle groups. Exercise intensity will be determined and monitored using the same method as the PG.

**Table 1:** Main characteristics and muscle groups when exercising with elastic bands

Characteristics	Muscle groups
Shoulder shrug	Trapezius
Straight arm shoulder abduction	Deltoid
Arm curls	Biceps
Chest press	Pectoralis major and deltoid anterior
Hip abduction	Mesoglutaeus
Stiff-legged dead lift	Hamstrings
Body squat	Gluteus maximus and quadriceps
Standing calf raise	Gastrocnemius and soleus

#### Pulmonary exercise combined with resistance exercise group

Participants in the PRG will continue with their prescribed medication treatment and receive 3 months of pulmonary exercise combined with exercising with resistance bands. The PRG regimen consists of two daily sessions, 7 days per week of pulmonary exercise and three sessions per week of exercising with resistance bands, over 3 months. On Sunday afternoons, that day’s exercises will be performed under the supervision and instruction of physiotherapists using a group format. Each session will span approximately 60 min and consist of (1) a 10-min warm-up exercise focusing on the flexibility of the peripheral skeletal muscle groups; (2) 20 min of prescribed pulmonary exercises like those done by the PG; (3) 5 min of rest, (4) 20 min of exercises with elastic bands like those done by the RG; and (5) a 5-min cool-down exercise focusing on stretching the peripheral skeletal muscle groups.

### Adherence

For patients in the CG, compliance will be quantified by the number of responses to telephone calls conducted once a week by the specialist, with compliance defined as no less than 85%. In other words, there should be no fewer than 11 responses. For patients in the three intervention groups, compliance will be quantified by the completion of the exercise log by patients and attendance at the supervised training sessions in the hospital. Compliance will be defined as no less than 85% of exercise sessions completed.

### Outcome measurement

All participants will undergo assessments within 1 week of being enrolled at baseline and at the end of 3 months. The primary outcomes are the pulmonary function test, 30-s arm curl test, and 6MWT. Secondary outcome measures include the Modified Medical Research Council Dyspnea Scale (mMRC), dual-energy X-ray absorptiometry (DXA), respiratory muscle strength test, peripheral skeletal muscle strength, sEMG, and questionnaires.

#### Assessments of basic characteristic

Assessments of basic characteristics mainly check pulmonary function, dyspnea, and body composition.

The pulmonary function test will be undertaken by specialist personnel. The test will be conducted according to American Thoracic Society / European Respiratory Society (ATS/ERS) standards [25]. Participants will be instructed to breathe, and three reproducible measurements each of FEV1, FVC, and maximal mid-expiratory flow will be obtained (Masterscreen-PFT, Jaeger, Germany). The highest value will be recorded and used for analysis.

The symptoms of dyspnea will be assessed using the mMRC, a five-point scale (0–4) of the severity of dyspnea, with a higher score indicating a higher severity [3].

Body composition will be assessed using DXA (DXA Prodigy, GE Lunar, USA). Patients will be instructed to remove metal objects from their clothing and body and lie on a test platform. DXA support software will calculate body composition including appendicular and body fat mass, appendicular and body skeletal muscle mass, and appendicular and body bone density. In addition, their body mass index will be calculated following measurement of their height and weight using digital scales (4643a, TANITA, Japan).

#### Muscle function tests

Assessments of muscle function mainly check respiratory muscle strength, and dominant upper and lower limb isokinetic muscle strength and sEMG.

Respiratory muscle strength, expressed as maximal inspiratory pressure and maximal expiratory pressure, will be assessed by specialist personnel following the ATS/ERS guidelines [26] using spirometry (Masterscreen-PFT, Jaeger, Germany). The maximal inspiratory pressure will be obtained when the subject exhales to the level of their residual functional volume, while the maximal expiratory pressure will be obtained when the subject inhales to the level of their total lung volume. Stable pressure readings will be recorded. The two measurements will be taken at least three times, and the highest result will be used for analysis. The interval between two consecutive measurements will be at least 1 min.

Peripheral skeletal muscle strength will be assessed using a biomechanical test and training system (CON-TREX, Physiomed, Germany). According to the user manual, subjects will be instructed to sit straight in a special chair, and the dominant limb muscle strength, including that of elbow and knee joints, will be evaluated. Straps will be applied across the chest and pelvis of the subject to prevent extraneous body movements during muscle contractions.

For the elbow joints, the alignment between the dynamometer rotational axis and the elbow joint’s rotational axis (humeral lateral condyle) will be checked before testing. The movement axis is parallel to the forearm and a strap will be used to maintain the connection. At the end of the movement, a hand will be placed to provide support in generating strength. After the subject is placed in a standard position, they will be instructed to perform flexions and extensions two or three times to determine the range of motion and for them to become familiar with the test.

For the knee joints, the alignment between the dynamometer rotational axis and the knee joint’s rotational axis (knee joint fibula capitulum) will be checked before the test. A resistance pad will be placed 2–3 cm above the ankle and a strap used to stabilize the dominant side. An obstacle will be used to prevent contraction of the disadvantaged limb. After the subject is placed in a standard position, they will be instructed to perform flexions and extensions two or three times to determine the range of motion and for them to become familiar with the test.

The test regimen for both elbow and knee joints consists of isokinetic concentric contractions. First, the subject will be asked to perform five continuous contractions at 60°/s of maximal flexion and extension, from which the computer will automatically calculate the maximum of five repetitions as the peak torque (N m) and the relative peak torque obtained by dividing the peak torque by the body weight (N m/kg). Then, the subject will be asked to perform 30 continuous contractions at 180°/s of maximal flexion and extension, from which the computer will automatically calculate the total work (joules) for the 30 repetitions. Verbal encouragement will be provided during testing to encourage maximum effort.

The peripheral skeletal muscles will be assessed by a wireless remote-sensing sEMG test and analysis system (Trigno™ Lab, Delsys, USA). The major muscles tested include the biceps and triceps in the dominant arm, and the rectus femoris, vastus medialis, and vastus lateralis in the dominant leg. The sEMG signal sampling rate will be set to 2000 Hz. In accordance with Surface Electromyography for the Non-Invasive Assessment of Muscles (SENIAM) (Simsek D, Different fatigue-resistant leg muscles and EMG response during whole-body vibration. Journal of Electromyography and Kinesiology. 2017, 37: 147-154) recommendations, the measurement sites will be prepared by shaving followed by cleaning the skin with alcohol. Surface electrodes will be placed at the highest point of the muscle belly during a contraction and parallel to the longitudinal arrangement of the muscle fibers. For the biceps and triceps, the sensor will be placed at the highest point of the muscle belly during flexion and extension. For the rectus femoris, the sensor will be placed midway between the anterior superior spine and the upper patella. For the vastus medialis, the sensor will be placed at 4/5 of the distance from the anterior superior spine to the medial joint of the knee. For the vastus lateralis, the sensor will be placed at the 2/3 of the distance from the anterior superior spine to the upper patella [27]. After positioning the electrodes, the experimental session will start with maximum voluntary isometric contractions measured by a MicroFET3 (Hoggan Scientific LLC, USA). The subjects will perform one trial to become familiar with the test and then perform two repetitions, each 5 s in duration. Verbal encouragement will be provided during the test to encourage maximum effort. There will be a 2-min rest between contractions.

#### Exercise capacity tests

To assess functional exercise capacity of the upper extremity, the 30-s arm curl test will be used according to the guidelines in the Senior Fitness Test Manual [28]. Participants will be instructed to sit on a chair with their back straight and feet flat on the floor. For women, a dumbbell weighing 5 lb. (2.3 kg) is used, and for men one weighing 8 lb. (3.6 kg). The weight will be held in the dominant hand, perpendicular to the floor. Participants will be instructed to move from the down position to the curled-up position without wrist movement. On the signal “go,” participants are to curl as many times as possible in 30 s.

To assess functional exercise capacity of the lower limb extremity, the 6MWT will be conducted following the ATS guidelines [29]. A 30-m straight line will be selected, and a chair placed at each end to signify the beginning and end of the testing area. Patients will be instructed to walk back and forth as quickly as possible over 6 min. At the start and end of the test, a modified Borg CR10 test will be used to assess the exercise intensity and dyspnea by the patients themselves. The test will be performed twice with a 30-min rest. After the test, the longest distance will be recorded and used for analysis.

#### Questionnaires

The questionnaires mainly assess quality of life, physical activity, and self-efficacy.

Health-related quality of life will be assessed by St. George’s Respiratory Questionnaire [30], which consists of three domains: symptoms (cough, sputum, asthma attack, etc.), activity (climbing, dressing, housework, etc.), and impact (anxiety, distress, insecurity, etc.). Scores range from 0 to 100, with higher scores reflecting a poorer quality of life.

Physical activity will be assessed by the Physical Activity Scale for the Elderly. The questionnaire asks about the physical activities performed in the last 7 days, and contains three domains: recreational physical activity, domestic physical activity, and occupational physical activity [31]. Questions are weighted and quantified at different levels, and higher scores reflect a higher level of physical activity.

Self-efficacy will be assessed by the Chronic Disease Self-Efficacy Scale, a six-item scale assessing confidence when performing certain activities. Higher scores indicate higher self-efficacy.

### Statistical analysis

SPSS version 23.0 will be used for data management and statistical analysis. A Kolmogorov–Smirnov test will be used to test for normality, and the normality data will be expressed as mean ± standard deviation, while skewed data will be expressed as median and interquartile range. An intention-to-treat analysis will be used for the allocation, irrespective of whether the participant completes the intervention. Missing outcome data will be handled using a mixed-model method. Then a per-protocol analysis will be conducted, which will include only subjects who completed all outcome measures and followed the intervention protocol. Comparisons of baseline differences between groups will be performed using one-way analysis of variance (ANOVA) or a non-parametric test. Training-related effects will be assessed using 2 × 2 ANOVA (group × time). Differences between variables over time (time effect, from baseline to 3 months) in each group will be evaluated using a paired *t*-test or a non-parametric test. For categorical variables, a chi-squared test will be employed to detect differences. A two-tailed *P* value of <0.05 will be considered to be significant. In addition, differing intervention effects on pulmonary function have been found in previous studies [4, 32, 33], and disease severity may be an important contributor. Therefore, a pre-specified subgroup analysis will be performed to investigate the differences in effectiveness according to disease severity.

### Safety

Supervised exercise will be performed with continuous heart rate monitoring. If the heart rate of a participant increases to ≥80% of their heart rate reserve, the exercise will be stopped. Any adverse effects including but not limited to dizziness, extreme breathlessness, extreme fatigue, chest pain, or falls during exercise or after a session will be documented and assessed by study staff to decide if the event is related to the exercise. In addition, subjects feeling discomfort while participating in the intervention will receive appropriate treatment.

### Data management

The medical records and data collected from participants will be stored securely. Only authorized research assistants will have access to the trial data.

## Discussion

The study expects to investigate the effects of prescribed pulmonary exercise on lung function, exercise capacity, skeletal muscle function, quality of life, and psychological function in stable COPD patients, and is expected to produce a comprehensive understanding of the effects of prescribed pulmonary exercise on COPD patients by comparing (1) usual care, (2) pulmonary exercise, (3) exercising with elastic bands, and (4) pulmonary exercise combined with exercising with elastic bands.

In terms of completion rate, a previous study based on a 6-month home-based *baduanjin* exercise intervention (including four sessions led by a trained therapist) in COPD patients (FEV1%pred: 37.12 ± 2.22) had a drop-out rate of 35% [11]. Another study of a *liuzijue* exercise intervention in COPD patients (FEV1%pred: 41.5 ± 4.5) with a similar protocol had a drop-out rate of 6% [8]. A 12-week home-based tai chi exercise intervention for COPD patients (FEV1%pred: 59 ± 16) had a drop-out rate of 10% [34]. The differences in the completion rate in previous studies may be attributed to various factors, including the duration of the intervention, the number of guidance sessions, differing content, and disease severity. Although the drop-out rates varied, a high completion rate for our traditional Chinese exercise intervention is predicted. In addition, drop-out rates for elastic band training protocols performed by COPD patients are in the range 15–25% for intervention protocols ranging in length from 8 to 12 weeks [19, 21, 22]. Hence, an assumed drop-out rate of 20% is feasible for this this study.

Dyspnea, the main complaint of COPD patients, can limit exercise capacity and impair skeletal muscle function, further aggravating dyspnea to form a vicious cycle [3]. Therefore, treating dyspnea and improving exercise capacity and skeletal muscle function in COPD patients are important.

As a gentle, self-healing, and functionally intact exercise, traditional Chinese exercise includes key characteristics of isometric contraction, stretching, relaxation, and adjustment of body posture combined with breathing techniques, with the main purpose of strengthening the body. A meta-analysis has shown that traditional Chinese exercise is a low-to-medium intensity exercise, suitable for middle-aged and elderly COPD patients with positive effects on lung function (indicated by improved FEV1 and FEV1/FVC), exercise capacity (6MWT) and quality of life [7]. A growing number of studies have attempted to explore the effects of this novel application of traditional Chinese exercise in preventing and controlling chronic diseases. Prescribed pulmonary exercise is based on the basic theory of traditional Chinese medicine and targeted to COPD rehabilitation using a reorganization of *liuzijue*, *wuqinxi*, *baduanjin*, and *yijinjing*. Liu et al. [12] found that rearranged traditional Chinese exercises could improve the activity of life and social participation significantly [12]. Since the study mainly focused on lung function, exercise capacity, activity of life, and inflammatory factors in COPD patients, the effects on skeletal muscle function and physical activity are unclear. Hence, this study aims to investigate the effects of prescribed pulmonary exercise in COPD patients using multidimensional indicators.

Exercising is the best available method of improving skeletal muscle function, and is considered as the cornerstone of PR for COPD [35]. Resistance exercise (achieved by various means, including with apparatus, bodyweight lifting, and elastic bands), as an important component of PR, can significantly improve skeletal muscle mass and function. It can be used to counterbalance skeletal muscle dysfunction [3]. Previous studies have found that resistance exercise (including with bands and conventional resistance exercise) has significant effects on improving fat-free body mass [19, 36] and skeletal muscle strength (assessed by digital dynamometry) in COPD. An additional benefit of improved exercise capacity (6MWT) was found when exercising with elastic bands compared with conventional resistance exercise [19]. An elastic band is made of natural latex, which is a cheap, lightweight, and portable material that can be safely applied by the subjects themselves. The use of bands is not limited by time or place. Hence, we wish to investigate the effects of exercising with elastic bands by COPD patients and compare it with the effects of prescribed pulmonary exercise, to clarify the specific effect and role of prescribed pulmonary exercise for stable COPD.

The main characteristic of exercising with resistance bands is an improvement in skeletal muscle function and exercise capacity by providing resistance to target muscle groups. Pulmonary exercise combines posture and breathing training, as a whole-body aerobic exercise, which may be effective in improving dyspnea and aerobic exercise capacity. It is possible that the combination of these two exercise modalities will have complementary effects in COPD patients, compared with a single exercise type. Hence, the study will combine prescribed pulmonary exercise with exercising with resistance bands for COPD patients, and compare the effects with each single exercise modality, to investigate this simple, convenient, home-based, and effective exercise for COPD patients.

There are several highlights to this study. First, the prescribed pulmonary exercise is a rearrangement of traditional Chinese exercise and targeted to COPD patients. The effects of this prescribed pulmonary exercise on lung function, respiratory muscle strength, exercise capacity, skeletal muscle function, quality of life, and physical activity on stable COPD patients will be evaluated. Second, the role of prescribed pulmonary exercise in improving skeletal muscle function and exercise capacity will be determined by comparing the effects of pulmonary exercise and an elastic band program. Third, the effect of pulmonary exercise combined with exercising with elastic bands in stable COPD patients will be determined, and the effects compared with a single program to determine whether combined exercise can achieve additional beneficial effects. Fourth, the study will apply comprehensive measurements to evaluate skeletal muscle function in patients with COPD, including isokinetic muscle strength testing, surface myoelectricity, 6MWT, and the 30-s arm curl test. Fifth, a sub-analysis will be performed to investigate if the treatment effects differ in patients with different disease severity to enable the optimization of treatment effects for all patients suffering from the troublesome disease. Finally, the study is in strict accordance with CONSORT guidelines, so that the effects of the prescribed pulmonary exercise can be clearly determined.

The study has some limitations. First, in any study with an exercise intervention, it is difficult to blind the therapists and participants, so there may be a placebo effect leading to biased results. Lack of blinding may also lead to a deviation from the intended intervention, with participants possibly seeking other treatments during the trial; however, methods such as keeping a log of exercise sessions and completing questionnaires on physical activity may be useful in identifying this. Second, the study mainly focuses on a home-based exercise intervention, and participant compliance may be lower than with a supervised outpatient intervention. However, designated staff will contact participants and supervise the extent of intervention through checking their exercise log books and by telephone. Third, the prescribed pulmonary exercise adopted in the study is a form of traditional Chinese exercise, so that knowledge about is restricted geographically, which may hinder its application and promotion. Finally, the participants included in the study are from the same hospital, so the study results will need to be confirmed by a multi-center study.

In conclusion, this article presents the design and protocol of a clinical trial investigating the effects of home-based prescribed pulmonary exercise in patients with stable COPD. The results of this study are expected to confirm the effects of prescribed pulmonary exercise in COPD patients and identify the strengths and shortcomings of prescribed pulmonary exercise, exercising with elastic bands, and a combined exercise program. In addition, the results may provide the basis for new, operable, effective, and home-based COPD PR programs.

### Trial status

The trial was registered in July 2018 and is currently in the recruitment phase.

## Additional file

Additional file 1: SPIRIT 2013 Checklist: Recommended items to address in a clinical trial protocol and related documents. (DOC 137 kb)

## Abbreviations

%pred: Percentage of the predicted value
6MWT: 6-min walking test
ANOVA: Analysis of variation
ATS/ERS: American Thoracic Society / European Respiratory Society
CG: Control group
COPD: Chronic obstructive pulmonary disease
DXA: Dual-energy X-ray absorptiometry
FEV1: Forced expiratory volume in 1 s
FVC: Forced vital capacity
GOLD: Global Initiative for Chronic Obstructive Lung Disease
mMRC: Modified Medical Research Council Dyspnea Scale
PASE: Physical Activity Scale for the Elderly
PG: Pulmonary exercise group
PR: Pulmonary rehabilitation
PRG: Pulmonary exercise and resistance exercise group
RG: Resistance exercise group
sEMG: Surface electromyography
SES: Chronic Disease Self-Efficacy Scale
SGRQ: St. George’s Respiratory Questionnaire
